# Combined analysis of circulating β-endorphin with gene polymorphisms in OPRM1, CACNAD2 and ABCB1 reveals correlation with pain, opioid sensitivity and opioid-related side effects

**DOI:** 10.1186/1756-6606-6-8

**Published:** 2013-02-12

**Authors:** Annica Rhodin, Alfhild Grönbladh, Harumi Ginya, Kent W Nilsson, Andreas Rosenblad, Qin Zhou, Mats Enlund, Mathias Hallberg, Torsten Gordh, Fred Nyberg

**Affiliations:** 1 Department of Surgical Sciences, University Hospital, Uppsala, SE, 75185, Sweden; 2 Department of Pharmaceutical Sciences, Division of Biological Research on Drug Dependence, Uppsala University, Uppsala, SE, 751 24, Sweden; 3 Precision System Science Co, Ltd 88, Kamihongo, Matsudo, Chiba, 270-0025, Japan; 4 Centre for Clinical Research, Central Hospital, Västerås, SE, 721 89, Sweden

**Keywords:** Chronic pain, Opioid sensitivity, Gene polymorphism, β-endorphin, mu-1-opioid peptide receptor (OPRM1), Calcium channel subunit 2 (CACNA2D2), ATP-binding cassette B1 (ABCB1)

## Abstract

**Background:**

Opioids are associated with wide inter-individual variability in the analgesic response and a narrow therapeutic index. This may be partly explained by the presence of single nucleotide polymorphisms (SNPs) in genes encoding molecular entities involved in opioid metabolism and receptor activation. This paper describes the investigation of SNPs in three genes that have a functional impact on the opioid response: OPRM1, which codes for the μ-opioid receptor; ABCB1 for the ATP-binding cassette B1 transporter enzyme; and the calcium channel complex subunit CACNA2D2. The genotyping was combined with an analysis of plasma levels of the opioid peptide β-endorphin in 80 well-defined patients with chronic low back pain scheduled for spinal fusion surgery, and with differential sensitivity to the opioid analgesic remifentanil. This patient group was compared with 56 healthy controls.

**Results:**

The plasma β-endorphin levels were significantly higher in controls than in pain patients.

A higher incidence of opioid-related side effects and sex differences was found in patients with the minor allele of the ABCB1 gene. Further, a correlation between increased opioid sensitivity and the major CACNA2D2 allele was confirmed. A tendency of a relationship between opioid sensitivity and the minor allele of OPRM1 was also found.

**Conclusions:**

Although the sample cohort in this study was limited to 80 patients it appears that it was possible to observe significant correlations between polymorphism in relevant genes and various items related to pain sensitivity and opioid response. Of particular interest is the new finding of a correlation between increased opioid sensitivity and the major CACNA2D2 allele. These observations may open for improved strategies in the clinical treatment of chronic pain with opioids.

## Background

The treatment of moderate to severe pain is largely dependent on the use of opioids [1]. Opioids have a narrow therapeutic index with wide variations in individual response [2]. Significant individual differences in sensitivity to these drugs can impair effective pain treatment and increase side effects. It is assumed that individual differences in opioid sensitivity may be due in part to genetic differences in the molecular elements involved in the pharmacokinetics and pharmacodynamics of opioids. Thus, genetic variability such as polymorphisms in the genes coding for opioid-metabolizing enzymes, transporter proteins, and the μ-opioid receptor could partly explain the observed inter-individual variations in response to opioids [3-5].

New molecular and genetic techniques have now made it possible to identify specific genes coding for pain-relevant proteins, genes that contain mutations that could explain the variations in pain response [6,7] as well as the differences in response to pain relieving drugs. Mutant mice and microarray studies have so far discovered approximately 200 protein entities thought to be involved in pain processing, and the genes coding for these proteins have been systematically investigated as candidate genes that may be relevant to pain perception [8]. Variations in these genes, single nucleotide polymorphisms (SNPs) or haplotypes (combination of alleles inherited together), are now being investigated in clinical populations (for review see [9]).

Since pain is a complex bio-psychosocial phenomenon, the task of searching for and finding single gene variations to explain individual differences could be seen as futile. However, in this study, the focus is on three genes, all of which encode proteins related to the pain-relieving effects of opioids, and on the endogenous opioid peptide β-endorphin. The search for candidate genes containing SNPs possibly associated with pain and opioid sensitivity resulted in the choice of the μ-opioid peptide receptor (*OPRM1*) gene [10-12], the ATP-binding cassette B1 (*ABCB1*) gene [13,14] and the calcium channel complex subunit (*CACNA2D2*) gene [8]. In the present study, the occurrence of SNPs in these genes was correlated with levels of circulating β-endorphin and clinical data from a well-characterized patient group with chronic low-back pain and differential sensitivity to the opioid remifentanil. The patients were classified as high responders, normal responders or non-responders to remifentanil by an intravenous opioid testing procedure (for details see Methods).

We chose to analyze an SNP at position 118 (A118G or 118A>G) of the gene for the *OPMR1* receptor, since this has been shown in earlier studies to alter the response to opioids [10]. The 118A>G mutation changes the base adenine to guanine, with the consequence that the amino acid asparagine is changed to aspartic acid at position 40 of the *OPMR1* amino acid sequence. As this change occurs on the extracellular part of the receptor, it results in an additional net charge with loss of a putative glucosylation site in the area of the ligand-receptor interaction. This can result in differences in opioid sensitivity, clinically manifested as altered analgesic requirements [15-17], variations in pain sensitivity [18], or altered propensity for opioid addiction [10]. The major allele is AA and, in this study, the minor allele was defined as the joint AG/GG allele, because of the low frequency of the homozygous minor allele GG.

The *ABCB1* gene is composed of 28 exons ranging in size from 49 to 209 base pairs, encoding an mRNA of 4.5 kb. The most common polymorphisms found in ABCB1 are 1236C>T, 2677 G>T/A/C, and 3435C>T. The transport protein P-glycoprotein, a product of the ABCB1 gene, plays an important role in the absorption and distribution of many drugs. P-glycoprotein is an ATPase-powered enzyme that transports substances, including opioids, across cell membranes and the blood–brain barrier [19,20], which regulates central nervous system exposure to drugs. It has been suggested that genetic variations in *ABCB1* could be a cause of inter-individual differences in drug response [21]. In this study, we investigated the C3435T SNP in exon 26 of *ABCB1*, where C>T. Thus, the major allele is CC, the heterozygous minor allele is CT and the homozygous minor allele is TT.

The *CACNA2D2* gene encodes a member of the alpha-2/delta subunit family, a protein in the voltage-dependent calcium channel complex [22]. Calcium channels mediate the influx of calcium ions into the cell upon membrane polarization and consist of a complex of alpha-1, alpha-2/delta, beta, and gamma subunits in a 1:1:1:1 ratio [22]. It has been demonstrated that this calcium channel interacts with the G-protein that mediates the effects of the μ-opioid receptor [23], with potential effects on pain and opioid requirements [24]. The α2δ fragment is also the effect site for gabapentin and pregabalin, drugs used for treating neuropathic pain [25]. The major allele is GG, the heterozygous allele is AG and the minor homozygous allele is AA [26].

β-endorphin is an opioid peptide produced primarily in the anterior lobe of the pituitary gland [27]. Following release from its precursor protein, pro-opiomelanocortin (POMC), β-endorphin is circulated via the blood stream to interact with specific opioid receptors located throughout the body [28]. The peptide interacts primarily with the μ-opioid peptide (MOP) receptor, although it can also bind to and activate other opioid receptors, e.g. the delta receptor [29]. It produces analgesia by inhibiting the firing of peripheral somatosensory fibers. Stress-induced increases in the release of β-endorphin are positively correlated with the amelioration of pain, whereas administration of exogenous opioids, such as fentanyl, reduces plasma levels of the peptide [27]. In experimental animals, exogenous opioids such as morphine have been shown to down-regulate the expression of POMC and subsequently induce a decrease in the biosynthesis of β-endorphin [30]. It has been suggested that decreased β-endorphin concentrations may play a role in a variety of chronic pain disorders [27].

Thus, in order to move the issue of the genetic aspects of chronic pain and opioid sensitivity a step forward, we investigated the influence of SNPs in the μ-receptor gene *OPRM1*, the *ABCB1* gene for the drug transporter P-glycoprotein and the calcium channel fragment gene α2δ *CACNAD2*, as well as the endogenous ligand for the μ-opioid receptor β-endorphin. These three candidate genes, all associated with pain processing and opioid analgesia, were analyzed in blood samples collected from 80 patients with chronic low back pain and 56 healthy controls. We also collected plasma samples for assessment of β-endorphin levels. The patients were classified as high responders (N=16), normal responders (N=44) or non-responders (N= 20) to the opioid remifentanil. All the study participants filled out an EORTC QLQ-30 Quality of Life (QoL) form for pain, function and symptom scoring.

## Results

The genotype frequencies of the minor allele of *OPRM1*, *ABCB1* and *CACNA2D2* are presented in Table 1, and were in accordance with Hardy-Weinberg equilibrium [31].

**Table 1:** Genotype frequencies

**Gene**	**SNP**	**High responder**	**Normal responder**	**Non responder**	**Control**
		N=16	N=44	N=20	N=56
OPRM1	A>G	44% N=7	22% N=8	25% N=5	25% N=14
ABCB1	C>T	32% N=5	27% N=12	25% N=5	34% N=19
CACNA2D2	G>A	0% N=0	27% N=12	30% N=6	25% N=14

Percentage of minor alleles of the OPMR1, ABCB1, and CACNA2D2 genes in high-, normal, non-responders and in control subjects. Due to the low frequency of the GG allele of OPRM1, the minor allele was defined as the combined AG/GG alleles. For further details see Methods and Results.

### β-endorphin levels

β-endorphin plasma levels were determined by radioimmunoassay (RIA) in patients and healthy controls (Table 2). The opioid responders had higher levels of β-endorphin (26.6 ± 3.59 fmol/ml) than the non-responders (24.7 ± 3.20 fmol/ml; p < 0.05) and the patients had lower β-endorphin levels (26.2 ± 3.57 fmol/ml) than the healthy controls (28.2 ± 4.63 fmol/ml; p < 0.01).

**Table 2:** Plasma levels of β-endorphin

	**N**	**β-endorphin fmol/L**	**p**
pain patients	80	26.2(3.59)	<0.05
controls	56	28.2(4.63)	
responder	60	26.6(3.57)	<0.01
non-responder	20	24.7(3.20)	
**Pain patients**			
male OPRM1 AA	31	26.8(3.25)	<0.05
male OPRM1 AG	8	24.2(2.96)	
female OPRM1 AA	28	26.1(4.02)	ns
female OPRM1 AG	11	25.9(3.50)	
**Controls**			
male OPRM1 AA	19	25.8(2.17)	ns
male OPRM1 AG	6	31.5(9.13)	ns
female OPRM1 AA	23	29.3(4.60)	ns
female OPRM1 AG	8	28.2(1.63)	ns

Plasma levels of the opioid peptide β-endorphin in patients with different opioid sensitivity. For further details see Methods and Results.

### OPRM1

The major and minor alleles of *OPRM1* were distributed as presented in Table 1, which shows an apparent higher incidence of the minor allele among the high responders than among the other participants. The genotype frequency of the minor allele was 7/16 (44%) in high responders, 8/44 (22%) in normal responders, 5/20 (25%) in non-responders, and 14/56 (25%) in controls (Table 2). The genotype frequency of the minor allele in controls (25%) corresponds to an allele frequency of 13.4% for the G allele in controls.

Male patients with the major allele of *OPRM1* had higher concentrations of β-endorphin (26.8 ± 3.25 fmol/ml) than male patients with the minor allele (24.2 ±2.96 fmol/ml; p < 0.05). There were no differences in that respect among female patients or in the control groups (Table 2). Furthermore, a borderline significant association was found in an age and sex adjusted model of OPRM1 and opioid sensitivity (r = 0.188, adjusted R^2^ = 0.035 p = 0.106).

### ABCB1

**Figure 1:**
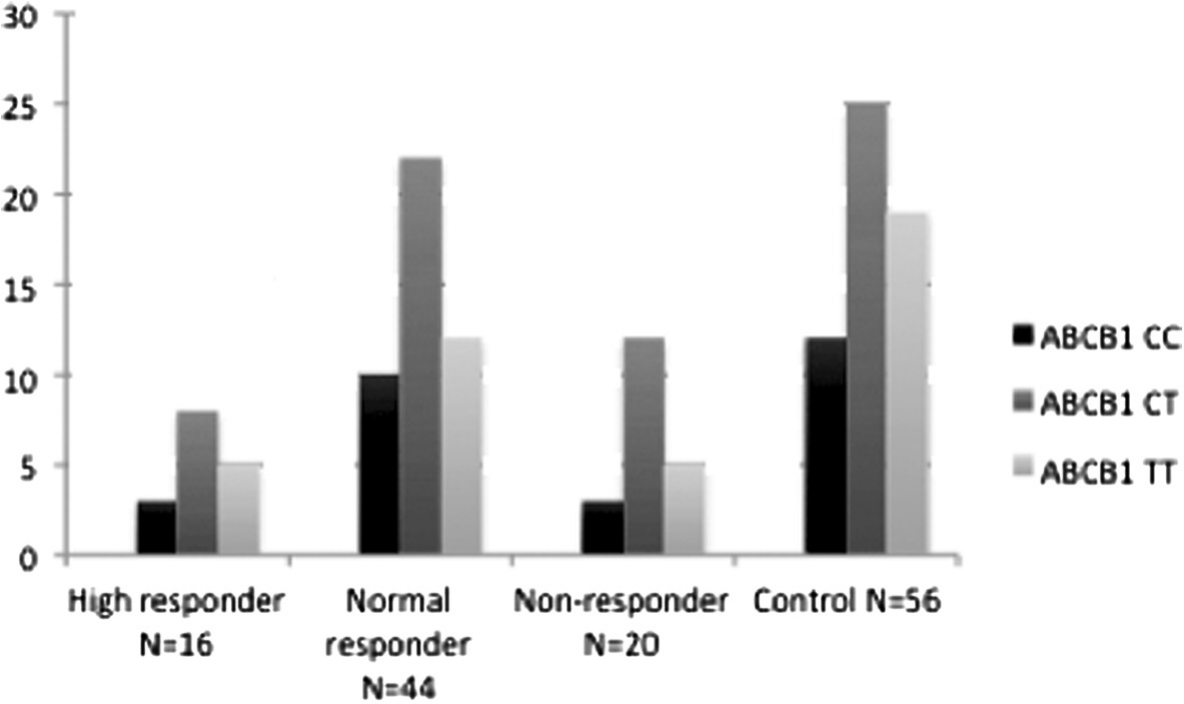
**Genotype frequency of ABCB1.** The genotype frequency of the ABCB1 SNP in chronic pain with different opioid sensitivity. For further details see Methods and Results.

*ABCB1* allelic distribution was similar in all groups (Figure 1). The frequency of the minor allele TT was 5/16 (31.75%) in high responders, 12/44 (27.2%) in normal responders, 5/20 (25%) in non-responders, and 19/56 (34%) in controls (Table 2).

**Figure 2:**
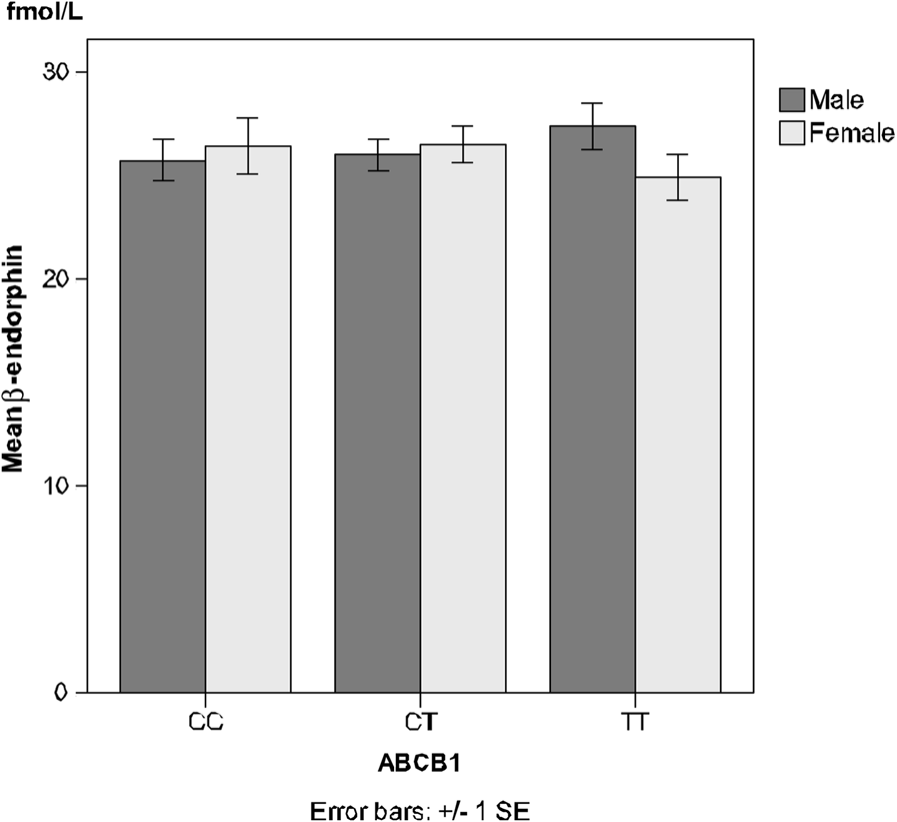
**β-endorphin levels and the ABCB1 SNP.** Plasma levels of the opioid peptide β-endorphin in males and females with different alleles of the ABCB1 SNP. A tendency towards sex difference (p = 0.057) in the group carrying the minor TT allele is indicated. For further details see Methods and Results. Values are presented as mean ± SEM.

There was a trend for different results among male and female patients regarding β-endorphin levels. Men with the minor allele TT tended to have higher β-endorphin levels (27.4 ± 3.40 fmol/L) than men with the major allele CC (25.8 ± 3.20 fmol/L). Similarly, women with the major allele CC tended to have higher β-endorphin levels (26.4 ± 3.30 fmol/L) than women with the minor TT allele (24.9 ± 3.66 fmol/L; Figure 2).

Among patients with the minor TT allele, there was a trend for sex differences in β-endorphin levels. Men tended to have higher levels than women (p = 0.057; Figure 2).

**Figure 3:**
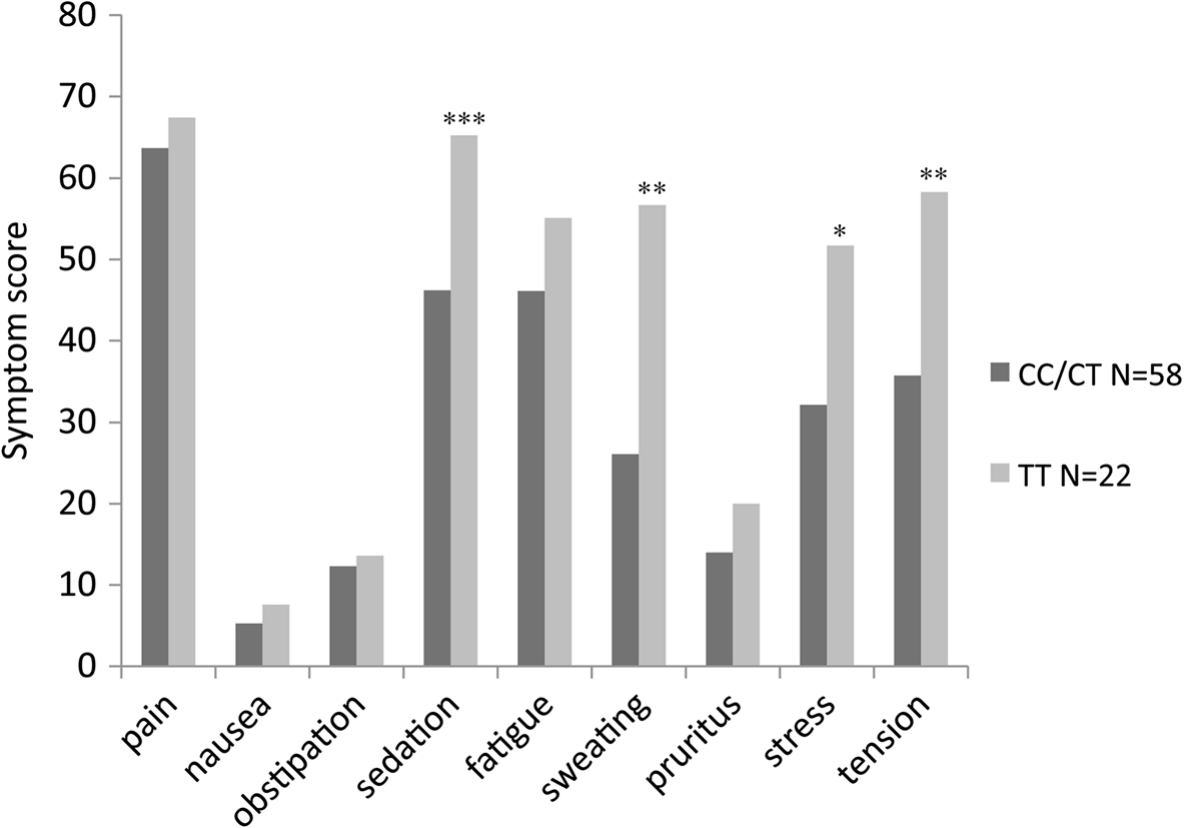
**Scores of pain and other symptoms.** Scores of pain and other symptoms for patients carrying the various ABCB1 alleles. For further details see Methods and Results.

**Figure 4:**
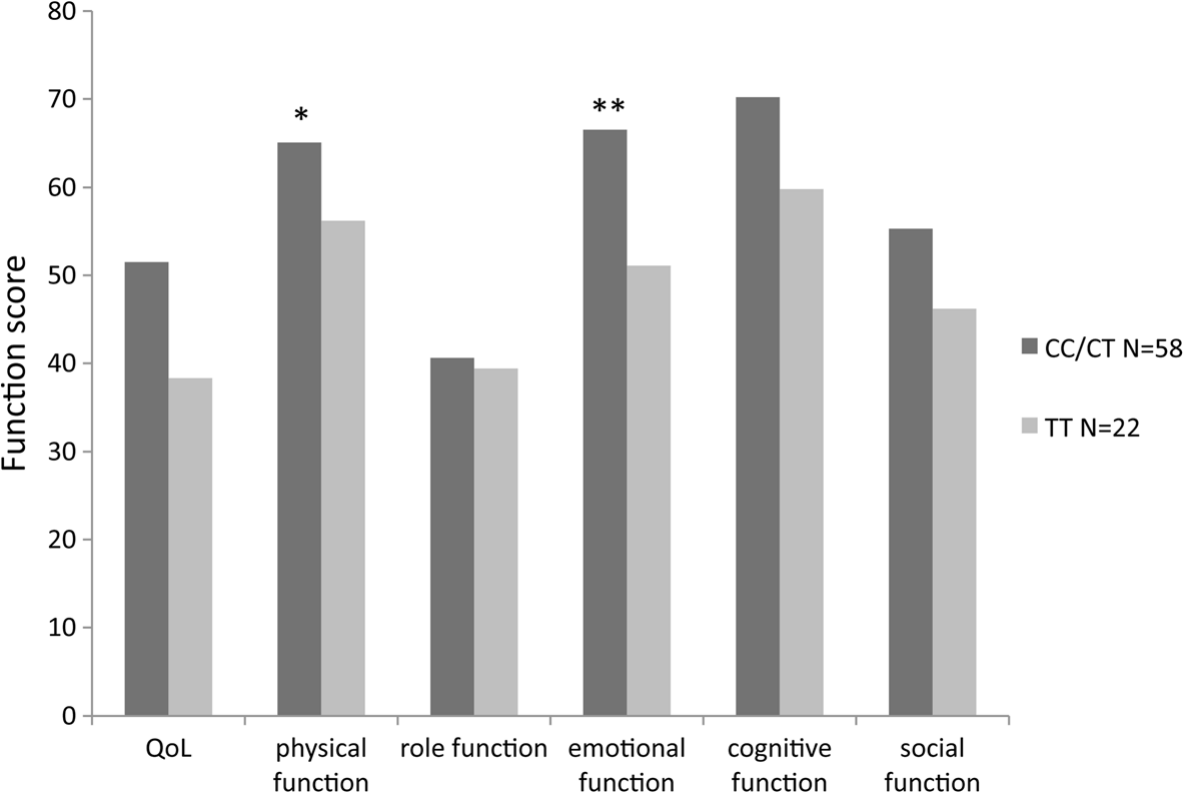
**Quality of Life and symptom scores.** Quality of Life (QoL) and symptom scores in patients carrying the various alleles of the ABCB1 gene. For further details see Methods and Results.

Patients with the minor TT allele also had more symptoms and side effects related to opioid treatment, such as sweating, sedation, tension and stress, than other patients (p < 0.05; Figure 3). The general quality of life and emotional function results were also poorer in the patients with the minor allele than in those with the CT/CC alleles. No difference in the rating of pain was noted between these groups, however (Figure 4).

### CACNA2D2

**Figure 5:**
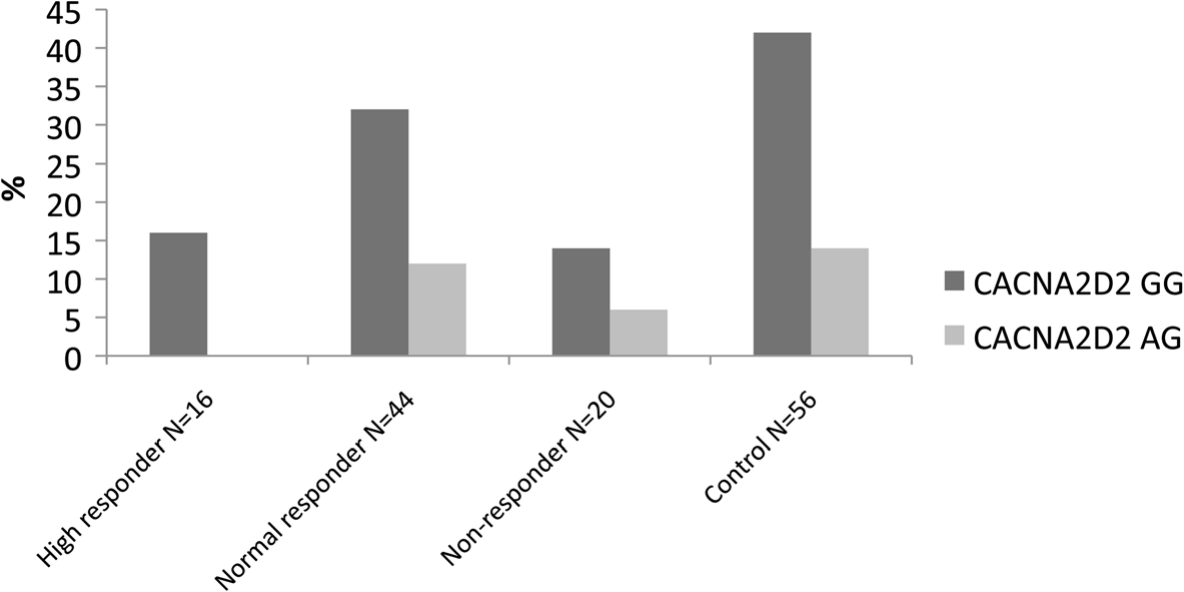
**The genotype frequency of CACNA2D2.** The genotype frequency of the CACNA2D2 gene in chronic pain patients with different opioid sensitivity. For further details see Methods and Results.

The *CACNA2D2* allelic distributions are presented in Figure 5. The minor *CACNA2D2* G>A allele was not found in any of the 16 high responders (0%), while 12/44 (27%) of the normal responder group, 6/20 (30%) of the non-responders, and 15/56 (25%) of the control group had this allele (Table 1). Thus, high responders to remifentanil had a higher incidence of the major *CACNA2D2* allele than normal responders, non-responders or controls (p < 0.05). There was also a trend for men with the major *CACNA2D2* allele to respond better to opioids than men with the minor allele (p = 0.087). In a multivariate model adjusted for age and sex there was a significant association between CACNA2D2 genotype and opioid sensitivity (r = 0.247, adjusted R^2^= 0.067, p = 0.029).

## Discussion

The use of opioids for the treatment of chronic pain has increased dramatically over the past decade [32,33]. However, as indicated above, the responsiveness to opioids in chronic pain patients may vary depending on individual differences but also on the type of pain [34,35]. For instance, a number of studies have evidenced that neuropathic pain patients are insensitive to opioids. The pharmacodynamic response to a given opioid depends on the nature of the receptor to which the opioid binds and its affinity for the receptor. During recent years, a wide range of candidate genes relevant to pain has been highlighted [8] and these genes are associated with a variety of molecules involved in pain processing, including neurotransmitters and their receptors and transporters, metabolic enzymes, ion channels, intracellular enzymes and second messengers. Minor changes in allelic variability, like SNPs, may affect the functionality of the encoded protein, resulting in an alteration of the protein activity or in the transcription rate leading to lower or higher amounts of the relevant protein [36]. An example of this is the decreased μ-opioid receptor mRNA and protein expression in human brain tissue in the presence of the *OPRM1* 118 G variant [37].

This study focused on the genes for the μ-opioid peptide (MOP) receptor, which mediates opioid effects, the ABCB1 transporter of opioids, and the calcium channel gene fragment α2δ, which affects the G-protein of the opioid receptor. The effects of these gene variants were in the present study measured in a group of patients with chronic pain and the effects were correlated with the perception of pain, the incidence of opioid side effects, plasma levels of β-endorphin and opioid sensitivity.

An important finding in the present study is that the plasma levels of β-endorphin the endogenous ligand for the MOP receptor were significantly lower in the chronic pain patients compared to control subjects. This observation agrees with previous reported findings showing that plasma levels of β-endorphin correlate inversely with pain levels in patients with various pain syndromes [38-42], i.e. the plasma concentration of this opioid peptide are lower in patients with poorly controlled pain but increase with pain relief [42]. Interestingly, in the present study opioid responders had higher plasma concentration of β-endorphin than non-responders.

Our results also demonstrate that high responders to remifentanil tended to be more likely to have the minor allele of *OPRM1* than normal responders, non-responders or controls. However, there were too few patients to confirm any statistical significance in this regard. The study reported by Landau et al. [15] indicating that women in labor who were homozygous for the minor *OPMR1* allele required less intrathecal fentanyl than those with the major allele is in accordance with our results. Similarly, Janicki and co-workers [43] demonstrated that patients with chronic pain who were homozygous for the minor allele used opioids less than those carrying the major allele. Furthermore, men with a higher pain threshold to experimental pressure pain were more likely to have the minor allele of A118G [18]. Similarly, according to a report by Huang et al. [44] women with the minor allele of the *OPRM1* receptor gene SNP IVS2+31 G>A have a higher pressure pain threshold than women with the major allele. Taken together, all these findings indicate that the minor alleles of the *OPRM1* gene could offer some protection from pain and subsequently have decreased requirement for opioids in chronic pain conditions.

In contrast, recent studies have indicated that patients with malignant disease who have the minor allele of *OPRM1* require more morphine than those with the major allele, and that patients who are undergoing abdominal hysterectomy [45] or total knee arthroplasty [46] who have the minor allele require larger intravenous doses of morphine postoperatively than those with the major allele. Also, a previous study has shown that fentanyl is less effective in subjects carrying the G allele of the OPRM1 A118G SNP than those with the A allele, and subjects with the G allele were fount to require more fentanyl for adequate postoperative pain control than those with the A allele [47]. Other studies suggest decreased sensitivity to the analgesic effects of opioids in persons with the minor allele and protection from opioid toxicity and side effects [48,49]. Thus, in patients with acute postoperative and cancer pain, those with the minor allele of *OPRM1* require more morphine than those with the major allele. However, the results of our study suggest that the situation could be different in patients with a phenotype of chronic pain from that in patients with postoperative or cancer pain and healthy subjects.

The consequence of the mutation A>G in the OPMR1 gene is an additional net charge with loss of a putative glucosylation site in the area of the ligand-receptor interaction and this feature may have consequences for the sensitivity to opioids of pain patients, although the phenotype of the pain seems to be essential.

Interestingly, the *OPRM1* A118G SNP has also been associated with higher heroin doses in addicted individuals [50]. In one of the first studies of the properties of the A118G allele, Bond et al. found a threefold increase in binding of β-endorphin [10], which could indicate that people with the minor allele might be at risk of opioid addiction. However, this finding has been disputed by Beyer and co-workers [51]. The men with chronic pain in our study who had the minor allele had lower β-endorphin levels than the men with the major allele, which could imply increased binding in the group with the minor allele.

It should be noted that in the present study we present data from the gene analysis as expressed in genotype frequency (see Tables 1, 2, 3) in contrast to many other investigators expressing their data by means of allele frequency. This may give the impression that we report a much higher frequency of the *OPRM1* A118G allele in our study population compared many other studies on the Caucasian population. However, calculation of the allele frequency in our material yields values that are in good agreement with those reported from other laboratories [46].

**Table 3:** Background data

	**Patients**	**Controls**
	n=80	n=56
High responder	16	
Normal responder	44	
Non-responder	20	
Sex M/F	39/41	25/31
Mean age (range)	46.2 (25-66)	40.5 (27-61)

Background data of the patients and controls participating in the study.

Our study also suggests that there may be differences between male and female patients regarding the effects of genetic variation in the *ABCB1* gene. Men with the minor TT allele had higher β-endorphin levels than men with the major CC allele but the reverse was true for women. Sia and co-workers [52] demonstrated a trend towards a higher risk of postoperative pain in women with the T-allele a difference underlying the potential importance of sex difference. Moreover, variants of the *ABCB1* gene may explain some portion of the inter-strain differences in opioid-induced hyperalgesia in mice and perhaps other consequences of chronic opioid administration [53].

Furthermore, patients homozygous for the minor TT allele of the ABCB1 gene experienced more opioid-related side effects such as sweating, muscular tension, stress and sedation than patients with the major CC/CT alleles. This is in agreement with other studies indicating an increased risk of opioid-induced side effects [54], such as early respiratory depression with fentanyl treatment [55]. It has been proposed that this effect involves impairment of P-glycoprotein transport, resulting in higher brain concentrations of the substrate (e.g. remifentanil). However, earlier studies have indicated decreased effects of methadone in patients with the minor TT allele [13,56] and a smaller increase in R-methadone levels with quetiapine [57]. The consequence of this mutation in the ABCB1 gene may be related to the effectiveness of the transporter P-glycoprotein, encoded by this gene. The transporter P-glycoprotein, is thus known to act on a broad range of prescription medicines, including opioids. The ABCB1_3435C>T SNP has been associated with mRNA, protein and serum levels, and with responses to a number of medical drugs [58].

An interesting observation made in this study is that the minor *CACNA2D2* G>A SNP was not found in any of the 16 high responders to remifentanil. This gene encodes a member of the alpha-2/delta subunit family, a protein in the voltage-dependent calcium channel complex. All the high responders to the opioid remifentanil had the major allele of *CACNA2D2*, suggesting an association between this allele and high opioid sensitivity. Interestingly, genetic variations of the Na^+^, K^+^, and Ca^2+−^channel genes have also been associated with migraine and neuropathic pain [59]. Very few, if any, study on opioid sensitivity in relation to the gene encoding the calcium channel fragment, *CACNA2D2* allele, has so far been reported. Interestingly, a recent study demonstrated an association between the voltage-gated calcium channels and the A118G OPRM1 polymorphism [60].

Thus, the effect of the mutation in the *CACNA2D2* G>A allele will certainly affect the effectiveness of the opioid. It is also essential for the action of β-endorphin in pain processing pathways. The link between β-endorphin and the calcium channel may be reflected by the observation that this opioid affects the inhibitory action of OPMR1 on the calcium channel expressed on pathways involved in nociception [60].

It is interesting to note that the low number of patients examined in this study was sufficient to provide some significant correlations in the group of patients with chronic pain, a complex bio-psychosocial entity. This was probably due to the homogeneity of the patient groups, and the fact that this study focused on correlating the presence of the gene variation affecting the function of the μ-opioid receptor with its ligand β-endorphin and opioid-related symptoms in a clinical population with the same chronic pain syndrome. This study also presents the gene for a calcium channel fragment as a plausible contributor to individual variations in opioid sensitivity. The sex variations were prominent, as has been demonstrated in other studies. These results take the issue of differences in opioid and pain sensitivity a step further; sex and genetic variations could explain differences in the pharmacokinetics and pharmacodynamics of opioids.

## Conclusions

This study confirms previously reported alterations in the levels of circulating β-endorphin in chronic pain. It further brings up the importance of genetic and sex factors in sensitivity to opioids. Thus, in this study we observed that the minor allele of the *OPRM1* gene tended to be associated with increased opioid sensitivity in chronic pain, that there were sex differences in β-endorphin levels and an increased incidence of opioid side effects in patients with the minor allele of the *ABCB1* transporter gene, and that there was a relationship between high opioid sensitivity and the presence of the major allele of the *CACNA2D2* gene.

## Methods

### Study population

Eighty patients with low back pain, who were scheduled for lumbar fusion surgery and had been investigated for opioid sensitivity, and 56 healthy volunteers were recruited for the study. The patient group comprised 39 men and 41 women with a mean age of 46.2 (25–66) years. The inclusion criteria were patients with chronic low back pain for more than 6 months on the waiting list for spinal fusion surgery. Age over 18 and ability to understand written and spoken Swedish was required. The patients were all scheduled for lumbar spinal fusion surgery after having performed diagnostic MR indicating degenerative disc disease, identification of diseased lumbar segment with discography and a clinical and neurological investigation by the orthopedic surgeon. At the time for the remifentanil study the pain of the patient was graded by visual analogue scale 0–100 mm. To be included in this study the patients were required to grade a minimum of 40 mm on this scale. The exclusion criteria were significant comorbidity that would preclude the planned operation. The control group consisted of 25 men and 31 women with a mean age of 40.5 (27–61) years. The ethnicity of the patients and the control population were mainly North European with the exception of 2 persons from each group that were of Middle Eastern heritage. Blood samples from the patients and healthy volunteers were collected and centrifuged. The red blood cells and plasma were used for DNA-analyses and for β-endorphin, respectively. Pain, QoL and opioid side effects and symptoms were measured in both patients and controls using the EORTC-OLQ-30 form. The rationale for using this instrument was that it is validated for opioid treated chronic pain and cancer patients [61,62]. Opioid side effects such as obstipation, nausea, sedation, sweating, dry mouth, pruritus, and symptoms such as perceived stress and muscular tension are evaluated in this instrument by the patients in the terms of none (1) a little (2), moderate (3) or severe disturbance (4). The numbers are then transcribed as occurrence between 0-100% as described in the above reference. The study was performed in compliance with the Helsinki Declaration and the regional Ethical Review Board in Uppsala approved the study. All patients and controls signed a written informed consent.

All patients had received opioids, but none was receiving daily doses of strong opioids at the time of opioid testing and blood sampling. The results obtained from the patients were compared with those from the control population of 56 healthy volunteers without pain.

### Test for opioid sensitivity with a target-controlled infusion of Remifentanil

The patients were investigated for opioid sensitivity using a target-controlled infusion of remifentanil, as described by Schraag et al. [63]. This was a double-blind, placebo-controlled investigation, where a significant response was taken to be a 50% reduction in pain, using a 100 mm visual analog scale, and a 50% increase in the pressure pain threshold at the point of maximum pain in the lower back. The pressure pain threshold was measured with an Algometer™ (Somedic AB, Sollentuna, Sweden) as described by Kosek et al. [64]. Basically, the instrument with a probe area of 1 cm^2^ and a rate of pressure increase of 50 kPa/s was applied to the area of maximal pain in the lumbar area and the patient indicated the point when pressure was experienced as pain; i.e. the pressure pain threshold. The target levels of remifentanil that were measured for 50% pain relief were 1–7 ng/ml. High responders to remifentanil had blood levels of the opioid in the range of 0.5–1.5 ng/ml, while normal responders required 2–7 ng/ml. In non-responders to remifentanil, the infusion had to be stopped because of sedation or side effects before any pain relief was obtained. There were 16 high responders, 44 normal responders and 20 non-responders.

### DNA amplification

The Magtration 12GC system (Precision System Science, Chiba, Japan) and the Magazorb® DNA Common Kit-200 (PSS, Chiba, Japan) were used for the total genomic DNA preparation from the whole blood samples. The DNA-fragments containing the SNP site was amplified in a reaction mixture on Mastercycler Ep Gradient S (Eppendorf AG, Germany).

The first PCR for *OPRM1* A118G were conducted using the TaKaRa LA Taq (TaKaRam Shiga, Japan) at 95°C for 3 min, then 30 cycles of 95°C for 30 s, 60°C for 30s and 72 for 1 min and hold at 4°C. PCR primers were forward 5’-CTGACGCTCCTCTCTGTCTCA-3’ and reverse 5’CAACATTGAGCCTTGGGAGT-3’. The second PCR were conducted with the identical program but using the HotGoldStar DNA polymerase (Eurogentec, Seraing, Belgium), using the primers forward 5’GAAAAGTCTCGGTGCTCCTG 3’ and reverse 5’ GCACACGATGGAGTAGAGGG 3’.

PCR for *CACNA2D2* was conducted with TaKaRa LA Taq polymerase (TaKaRa, Shiga, Japan) by touch-down PCR: 94°C for 3 min, then 10 cycles of 94°C for 20 s, 60°C down to 50°C (−1°C/cycle) for 30s, 72°C for 1 min, then 25 cycles of 94°C for 20 s, 50°C for 30 s, 72°C for 1 min, then 72°C for 10 min and hold at 4°C. Primers used were forward 5’-AAGACGGATGGCCTCGTTA-3’ and reverse 5’-ACATATGGATGGCCAGTTGAA-3’. The second PCR was conducted using the HotGoldStar DNA polymerase (Eurogentec, Seraing, Belgium) at 94°C for 3 min, then 40 cycles of 94°C for 30 s, 65°C for 30 s, 72°C for 1 min and ending with 72°C for 1 min and hold at 4°C, using primers forward 5’-TCCAACATCACTCGGGCCAACT-3’ and reverse 5’-TTGTTGGCACAGGCCATCCACT-3’.

Amplification of *ABCB1* was conducted with one PCR at 94°C, 2 min, 94°C 30 sec, 60°C 30 sec, 68°C 1 min 30 cycles, 4°C hold using the AccuPrime Taq DNA polymerase (Invitrogen, Carlsbad, CA). Primers used were forward 5’-GAGCCCATCCTGTTTGACTG-3’ and reverse 5’-ACTATAGGCCAGAGAGGCTGC-3´.

The PCR product was purified with the PCR Clean-Up Kit (Invitrogen, Carlsbad, CA) using the Magtration 12GC system and the concentration were determined using NanoDrop (Long beach, CA, USA).

### SNP genotyping

The Handy Bio-Strand method was used for the SNP genotyping of *OPRM1* A118G (rs1799971), as described earlier [65]. Briefly, the amplified DNA was spotted on a micro-porous nylon thread (Bio-Strand) and hybridized with allele-specific oligonucleotide competitive hybridization (ASOCH). The Cy5 oligonucleotide Cy5-Tag1 was used as a landmark. The sequences of the positive controls, used to confirm the SNP genotyping of *OPRM1* A118G by ASOCH, were as follows: 5’ GTCGGACAGGTTGCCATC TAAGT 3’ (AA), 5’ GTCGGACAGGTCGCCATCTAAGT 3’ (GG). Hybridization was conducted automatically in room temperature using the Magtration 12GC System. The Bio-Strand Tip was first pre-hybridized by incubation in 450 μl of a solution containing 2xSSC (1xSSC is 150 mM NaCl and 15 mM sodium citrate), 0.1% SDS and 200 μl/ml salmon sperm DNA (Invitrogen, Carlsbad, CA), then incubated 5 min in 450 μl of the hybridization solution containing 2xSSC, 0.1% SDS, 200 μl/ml salmon sperm DNA and 10nM Cy5 probes. The Bio-strand Tip was washed for 2 min each in 450 μl washing buffer 1 (2xSSC and 0.1% SDS) washing buffer 2 (1xSSC and 0.1 %SDS), washing buffer 3 (0.1xSSC and 0.1% SDS) and then soaked in 450 μl of 2xSSC. The Cy5 probes for ASOCH were: 5’-ATGGCGACCTG 3’ (G), 5’ Cy5-ATGGCAACCTG 3’ (A). Two non-labeled oligonucleotides were used as competitor to the Cy5-probes, (5’-ATGG CAACCTG-3’) for G and (5’-ATGGCGACCTG-3’) for A. The Cy5 fluorescent signal was scanned with the Handy Bio-Strand scanner (PSS, Chiba, Japan) and the results were analyzed with Hy-Soft software (PSS, Chiba, Japan). In addition, all OPRM1 SNP were confirmed by PCR direct sequencing.

PCR direct sequencing was used to analyse *CACNA2D2* G>A (rs5030977) and *ABCB1* C3435T (rs1045642) SNP genotypes. Briefly, the PCR-product was mixed with Exo-SAP-IT (USB, Cleveland, OH, USA), diluted 1:20 with H2O and incubated in 37°C, 30 min. Exo-SAP-IT was inactivated by heating at 80°C for 20 min. Direct DNA sequencing were performed at Macrogen (Seoul, Korea).

### Radioimmunoassay

The frozen plasma samples were thawed on ice and centrifuged at 4°C for 10 min at 3000 × g. The supernatants were collected, diluted (1:5) with 0.1 M formic acid and 0.018 M pyridine (buffer I) and separated on minicolumns (1 ml) packed with SP-Sephadex C-25 gel according to a previously outlined procedure [66]. The columns were washed with 10 ml buffer I and then, after application of the sample, washed with additional 5 ml of 0.1 M formic acid/0.1 M pyridine, pH 4.4 (buffer II). The peptide-containing fractions were then eluted with 4 ml of 1.6 M formic acid/1.6 M pyridine, pH 4.4 (buffer V). All buffers contained 0.01% mercaptoethanol. The eluted samples were then evaporated in a Speed Vac centrifuge (Savant, Hicksville, NY, USA).

The EURIA-β-Endorphin kit (EURO-DIAGNOSTICA AB, Sweden) was used for the β-endorphin radioimmunoassay (RIA). This RIA is based on the principle of double-antibody precipitation. The evaporated samples were diluted with 220 μl diluent (0.05 M phosphate, pH 7.4, 0.25% human serum albumin, 0.05% sodium azide, 0.25% EDTA and 500 KIU Trasylol®/ml) and incubated with 100 μl of anti-β-endorphin antiserum for 24 h at 4°C. After incubation, the labeled peptide was added to each sample and incubated for an additional 24 h, at 4°C. Thereafter, the double antibody PEG was added, and the test tubes were incubated for 60 min and then centrifuged for 15 min at 3000 × g at 4°C. Finally, the supernatants were decanted and the radioactivity of the precipitates was counted in a gamma counter.

### Statistical methods

The material was analyzed with SPSS 14 quantitative data using Student’s t-tests. Ordinal data and variables not normally distributed were analyzed by the Mann–Whitney U-test, the Kruskal Wallis test and the z-test for comparison of population proportions. Hardy-Weinberg equilibrium was assessed for each SNP by χ2 analyses. The findings in univariate analyses were furthermore investigated in multivariate models adjusted for age and sex.

## Competing interests

Harumi Ginya, working as a guest researcher at Uppsala University (Dpt. of Pharmaceutical Biosciences, Sweden) when this project was conducted, is employed at PSS, Tokyo, Japan, from where the Magtration 12GC system was bought. The remaining authors declare no competing interests.

## Authors’ contributions

AR participated in the contact with the patients, collected samples, analyzed the data and drafted the manuscript. AG participated in the SNP genotyping, the radioimmunoassay and helped to draft the manuscript. HG carried out the SNP analysis. KWN and AR performed the statistical analysis. QZ carried out the radioimmunoassay. ME participated in opioid sensitivity testing and analysis. MH participated in the design of the study. TG participated in the design and coordination of the study and helped to draft the manuscript. FN conceived of the study, participated in the design and coordination and drafted the manuscript. All authors read and approved the final manuscript.

## References

[B1] Furlan AD Reardon R Weppler C Opioids for chronic noncancer pain: a new Canadian practice guideline CMAJ 2010 182 923 930 20439443 10.1503/cmaj.100187 PMC2882451

[B2] Angst MS Phillips NG Drover DR Tingle M Ray A Swan GE Lazzeroni LC Clark JD Pain sensitivity and opioid analgesia: a pharmacogenomic twin study Pain 2012 153 1397 1409 10.1016/j.pain.2012.02.022 22444188 PMC3377769

[B3] Ikeda K Ide S Han W Hayashida M Uhl GR Sora I How individual sensitivity to opiates can be predicted by gene analyses Trends Pharmacol Sci 2005 26 311 317 10.1016/j.tips.2005.04.001 15925706

[B4] Somogyi AA Barratt DT Coller JK Pharmacogenetics of opioids Clin Pharmacol Ther 2007 81 429 444 10.1038/sj.clpt.6100095 17339873

[B5] Bayerer B Stamer U Hoeft A Stuber F Genomic variations and transcriptional regulation of the human mu-opioid receptor gene Eur J Pain 2007 11 421 427 10.1016/j.ejpain.2006.06.004 16843022

[B6] Diatchenko L Anderson AD Slade GD Fillingim RB Shabalina SA Higgins TJ Sama S Belfer I Goldman D Max MB Three major haplotypes of the beta2 adrenergic receptor define psychological profile, blood pressure, and the risk for development of a common musculoskeletal pain disorder Am J Med Genet B Neuropsychiatr Genet 2006 141B 449 462 10.1002/ajmg.b.30324 16741943 PMC2570772

[B7] Mogil JS McCarson KE Identifying pain genes: bottom-up and top-down approaches J Pain 2000 1 66 80 10.1054/jpai.2000.9821 14622845

[B8] Belfer I Wu T Kingman A Krishnaraju RK Goldman D Max MB Candidate gene studies of human pain mechanisms: methods for optimizing choice of polymorphisms and sample size Anesthesiology 2004 100 1562 1572 10.1097/00000542-200406000-00032 15166579

[B9] Fernandez Robles CR Degnan M Candiotti KA Pain and genetics Curr Opin Anaesthesiol 2012 25 444 449 10.1097/ACO.0b013e3283556228 22732422

[B10] Bond C LaForge KS Tian M Melia D Zhang S Borg L Gong J Schluger J Strong JA Leal SM Single-nucleotide polymorphism in the human mu opioid receptor gene alters beta-endorphin binding and activity: possible implications for opiate addiction Proc Natl Acad Sci USA 1998 95 9608 9613 10.1073/pnas.95.16.9608 9689128 PMC21386

[B11] Befort K Filliol D Decaillot FM Gaveriaux-Ruff C Hoehe MR Kieffer BL A single nucleotide polymorphic mutation in the human mu-opioid receptor severely impairs receptor signaling J Biol Chem 2001 276 3130 3137 10.1074/jbc.M006352200 11067846

[B12] Sia AT Lim Y Lim EC Goh RW Law HY Landau R Teo YY Tan EC A118G single nucleotide polymorphism of human mu-opioid receptor gene influences pain perception and patient-controlled intravenous morphine consumption after intrathecal morphine for postcesarean analgesia Anesthesiology 2008 109 520 526 10.1097/ALN.0b013e318182af21 18719451

[B13] Coller JK Barratt DT Dahlen K Loennechen MH Somogyi AA ABCB1 genetic variability and methadone dosage requirements in opioid-dependent individuals Clin Pharmacol Ther 2006 80 682 690 10.1016/j.clpt.2006.09.011 17178268

[B14] Campa D Gioia A Tomei A Poli P Barale R Association of ABCB1/MDR1 and OPRM1 gene polymorphisms with morphine pain relief Clin Pharmacol Ther 2008 83 559 566 10.1038/sj.clpt.6100385 17898703

[B15] Landau R Kern C Columb MO Smiley RM Blouin JL Genetic variability of the mu-opioid receptor influences intrathecal fentanyl analgesia requirements in laboring women Pain 2008 139 5 14 10.1016/j.pain.2008.02.023 18403122 PMC2669083

[B16] Reyes-Gibby CC Shete S Rakvag T Bhat SV Skorpen F Bruera E Kaasa S Klepstad P Exploring joint effects of genes and the clinical efficacy of morphine for cancer pain: OPRM1 and COMT gene Pain 2007 130 25 30 10.1016/j.pain.2006.10.023 17156920 PMC1995596

[B17] Klepstad P Rakvag TT Kaasa S Holthe M Dale O Borchgrevink PC Baar C Vikan T Krokan HE Skorpen F The 118 A > G polymorphism in the human mu-opioid receptor gene may increase morphine requirements in patients with pain caused by malignant disease Acta Anaesthesiol Scand 2004 48 1232 1239 10.1111/j.1399-6576.2004.00517.x 15504181

[B18] Fillingim RB Kaplan L Staud R Ness TJ Glover TL Campbell CM Mogil JS Wallace MR The A118G single nucleotide polymorphism of the mu-opioid receptor gene (OPRM1) is associated with pressure pain sensitivity in humans J Pain 2005 6 159 167 10.1016/j.jpain.2004.11.008 15772909

[B19] Wandel C Kim R Wood M Wood A Interaction of morphine, fentanyl, sufentanil, alfentanil, and loperamide with the efflux drug transporter P-glycoprotein Anesthesiology 2002 96 913 920 10.1097/00000542-200204000-00019 11964599

[B20] Mizutani T Masuda M Nakai E Furumiya K Togawa H Nakamura Y Kawai Y Nakahira K Shinkai S Takahashi K Genuine functions of P-glycoprotein (ABCB1) Curr Drug Metab 2008 9 167 174 10.2174/138920008783571756 18288958

[B21] Schwab M Eichelbaum M Fromm MF Genetic polymorphisms of the human MDR1 drug transporter Annu Rev Pharmacol Toxicol 2003 43 285 307 10.1146/annurev.pharmtox.43.100901.140233 12359865

[B22] Gao B Sekido Y Maximov A Saad M Forgacs E Latif F Wei MH Lerman M Lee JH Perez-Reyes E Functional properties of a new voltage-dependent calcium channel alpha(2)delta auxiliary subunit gene (CACNA2D2) J Biol Chem 2000 275 12237 12242 10.1074/jbc.275.16.12237 10766861 PMC3484885

[B23] Currie KP G protein modulation of CaV2 voltage-gated calcium channels Channels (Austin) 2010 4 497 509 21150298 10.4161/chan.4.6.12871 PMC3052249

[B24] Cao YQ Voltage-gated calcium channels and pain Pain 2006 126 5 9 10.1016/j.pain.2006.10.019 17084979

[B25] Alden KJ Garcia J Differential effect of gabapentin on neuronal and muscle calcium currents J Pharmacol Exp Ther 2001 297 727 735 11303064

[B26] Angeloni D Wei MH Duh FM Johnson BE Lerman MI A G-to-A single nucleotide polymorphism in the human alpha 2 delta 2 calcium channel subunit gene that maps at chromosome 3p21.3 Mol Cell Probes 2000 14 53 54 10.1006/mcpr.1999.0277 10722793

[B27] Hartwig AC Peripheral beta-endorphin and pain modulation Anesth Prog 1991 38 75 78 1814247 PMC2161980

[B28] Goldfarb AH Jamurtas AZ Beta-endorphin response to exercise an update Sports Med 1997 24 8 16 10.2165/00007256-199724010-00002 9257407

[B29] Aloyo VJ Pazdalski PS Evidence that beta-endorphin is an agonist at bovine pineal delta-opioid receptors Eur J Pharmacol 1995 288 295 301 10.1016/0922-4106(95)90041-1 7774673

[B30] Fang Y Kelly MJ Ronnekleiv OK Proopiomelanocortin (POMC) mRNA expression: distribution and region-specific down-regulation by chronic morphine in female guinea pig hypothalamus Brain Res Mol Brain Res 1998 55 1 8 10.1016/S0169-328X(97)00348-3 9645954

[B31] Guo SW Thompson EA Performing the exact test of Hardy-Weinberg proportion for multiple alleles Biometrics 1992 48 361 372 10.2307/2532296 1637966

[B32] Boudreau D Von Korff M Rutter CM Saunders K Ray GT Sullivan MD Campbell CI Merrill JO Silverberg MJ Banta-Green C Weisner C Trends in long-term opioid therapy for chronic non-cancer pain Pharmacoepidemiol Drug Saf 2009 18 1166 1175 10.1002/pds.1833 19718704 PMC3280087

[B33] Fredheim OM Skurtveit S Breivik H Borchgrevink PC Increasing use of opioids from 2004 to 2007 - pharmacoepidemiological data from a complete national prescription database in Norway Eur J Pain 2010 14 289 294 10.1016/j.ejpain.2009.05.006 19505834

[B34] Arner S Meyerson BA Lack of analgesic effect of opioids on neuropathic and idiopathic forms of pain Pain 1988 33 11 23 10.1016/0304-3959(88)90198-4 2454440

[B35] Smith HS Meek PD Pain responsiveness to opioids: central versus peripheral neuropathic pain J Opioid Manag 2011 7 391 400 10.4113/jom.2011.1153 22165038

[B36] Yan H Yuan W Velculescu VE Vogelstein B Kinzler KW Allelic variation in human gene expression Science 2002 297 1143 10.1126/science.1072545 12183620

[B37] Zhang Y Wang D Johnson AD Papp AC Sadee W Allelic expression imbalance of human mu opioid receptor (OPRM1) caused by variant A118G J Biol Chem 2005 280 32618 32624 10.1074/jbc.M504942200 16046395

[B38] Lopez JA Peran F Altuzarra A Garrido F Arjona V Plasmatic beta-endorphin levels and thalamic surgery for pain Neurol Res 1985 7 35 38 2860587 10.1080/01616412.1985.11739698

[B39] Mystakidou K Befon S Hondros K Kouskouni E Vlahos L Continuous subcutaneous administration of high-dose salmon calcitonin in bone metastasis: pain control and beta-endorphin plasma levels J Pain Symptom Manage 1999 18 323 330 10.1016/S0885-3924(99)00081-0 10584455

[B40] Facchinetti F Nappi G Savoldi F Genazzani AR Primary headaches: reduced circulating beta-lipotropin and beta-endorphin levels with impaired reactivity to acupuncture Cephalalgia 1981 1 195 201 10.1046/j.1468-2982.1981.0104195.x 6290071

[B41] Leonard TM Klem SA Asher MA Rapoff MA Leff RD Relationship between pain severity and serum beta-endorphin levels in postoperative patients Pharmacotherapy 1993 13 378 381 8361864

[B42] El-Sheikh N Boswell MV Plasma Beta-endorphin levels before and after relief of cancer pain Pain Physician 2004 7 67 70 16868614

[B43] Janicki PK Schuler G Francis D Bohr A Gordin V Jarzembowski T Ruiz-Velasco V Mets B A genetic association study of the functional A118G polymorphism of the human mu-opioid receptor gene in patients with acute and chronic pain Anesth Analg 2006 103 1011 1017 10.1213/01.ane.0000231634.20341.88 17000822

[B44] Huang CJ Liu HF Su NY Hsu YW Yang CH Chen CC Tsai PS Association between human opioid receptor genes polymorphisms and pressure pain sensitivity in females* Anaesthesia 2008 63 1288 1295 10.1111/j.1365-2044.2008.05760.x 19032295

[B45] Chou WY Wang CH Liu PH Liu CC Tseng CC Jawan B Human opioid receptor A118G polymorphism affects intravenous patient-controlled analgesia morphine consumption after total abdominal hysterectomy Anesthesiology 2006 105 334 337 10.1097/00000542-200608000-00016 16871067

[B46] Chou WY Yang LC Lu HF Ko JY Wang CH Lin SH Lee TH Concejero A Hsu CJ Association of mu-opioid receptor gene polymorphism (A118G) with variations in morphine consumption for analgesia after total knee arthroplasty Acta Anaesthesiol Scand 2006 50 787 792 10.1111/j.1399-6576.2006.01058.x 16879459

[B47] Fukuda K Hayashida M Ikeda K [Postoperative pain management following orthognathic surgery in consideration of individual differences--is the antinociceptive effect of fentanyl related to the genotype involving nucleotide at OPRM1?] Masui 2009 58 1102 1108 19764432

[B48] Lotsch J Skarke C Grosch S Darimont J Schmidt H Geisslinger G The polymorphism A118G of the human mu-opioid receptor gene decreases the pupil constrictory effect of morphine-6-glucuronide but not that of morphine Pharmacogenetics 2002 12 3 9 10.1097/00008571-200201000-00002 11773859

[B49] Lotsch J Zimmermann M Darimont J Marx C Dudziak R Skarke C Geisslinger G Does the A118G polymorphism at the mu-opioid receptor gene protect against morphine-6-glucuronide toxicity? Anesthesiology 2002 97 814 819 10.1097/00000542-200210000-00011 12357145

[B50] Shi J Hui L Xu Y Wang F Huang W Hu G Sequence variations in the mu-opioid receptor gene (OPRM1) associated with human addiction to heroin Hum Mutat 2002 19 459 460 11933204 10.1002/humu.9026

[B51] Beyer A Koch T Schroder H Schulz S Hollt V Effect of the A118G polymorphism on binding affinity, potency and agonist-mediated endocytosis, desensitization, and resensitization of the human mu-opioid receptor J Neurochem 2004 89 553 560 10.1111/j.1471-4159.2004.02340.x 15086512

[B52] Sia AT Sng BL Lim EC Law H Tan EC The influence of ATP-binding cassette sub-family B member −1 (ABCB1) genetic polymorphisms on acute and chronic pain after intrathecal morphine for caesarean section: a prospective cohort study Int J Obstet Anesth 2010 19 254 260 10.1016/j.ijoa.2010.03.001 20627697

[B53] Liang DY Liao G Lighthall GK Peltz G Clark DJ Genetic variants of the P-glycoprotein gene Abcb1b modulate opioid-induced hyperalgesia, tolerance and dependence Pharmacogenet Genomics 2006 16 825 835 10.1097/01.fpc.0000236321.94271.f8 17047491

[B54] Coulbault L Beaussier M Verstuyft C Weickmans H Dubert L Tregouet D Descot C Parc Y Lienhart A Jaillon P Becquemont L Environmental and genetic factors associated with morphine response in the postoperative period Clin Pharmacol Ther 2006 79 316 324 10.1016/j.clpt.2006.01.007 16580900

[B55] Park HJ Shinn HK Ryu SH Lee HS Park CS Kang JH Genetic polymorphisms in the ABCB1 gene and the effects of fentanyl in Koreans Clin Pharmacol Ther 2007 81 539 546 10.1038/sj.clpt.6100046 17192767

[B56] Li Y Kantelip JP Gerritsen-van Schieveen P Davani S Interindividual variability of methadone response: impact of genetic polymorphism Mol Diagn Ther 2008 12 109 124 18422375 10.1007/BF03256276

[B57] Uehlinger C Crettol S Chassot P Brocard M Koeb L Brawand-Amey M Eap CB Increased (R)-methadone plasma concentrations by quetiapine in cytochrome P450s and ABCB1 genotyped patients J Clin Psychopharmacol 2007 27 273 278 10.1097/JCP.0b013e3180592ad2 17502774

[B58] Loeuillet C Weale M Deutsch S Rotger M Soranzo N Wyniger J Lettre G Dupre Y Thuillard D Beckmann JS Promoter polymorphisms and allelic imbalance in ABCB1 expression Pharmacogenet Genomics 2007 17 951 959 10.1097/FPC.0b013e3282eff934 18075465

[B59] Estevez M Gardner KL Update on the genetics of migraine Hum Genet 2004 114 225 235 10.1007/s00439-003-1055-9 14624354

[B60] Lopez Soto EJ Raingo J A118G Mu Opioid Receptor polymorphism increases inhibitory effects on CaV2.2 channels Neurosci Lett 2012 523 190 194 10.1016/j.neulet.2012.06.074 22796651

[B61] Wincent A Liden Y Arner S Pain questionnaires in the analysis of long lasting (chronic) pain conditions Eur J Pain 2003 7 311 321 10.1016/S1090-3801(03)00044-2 12821401

[B62] Hjermstad MJ Fayers PM Bjordal K Kaasa S Health-related quality of life in the general Norwegian population assessed by the European Organization for Research and Treatment of Cancer Core Quality-of-Life Questionnaire: the QLQ=C30 (+ 3) J Clin Oncol 1998 16 1188 1196 9508207 10.1200/JCO.1998.16.3.1188

[B63] Schraag S Kenny GN Mohl U Georgieff M Patient-maintained remifentanil target-controlled infusion for the transition to early postoperative analgesia Br J Anaesth 1998 81 365 368 10.1093/bja/81.3.365 9861121

[B64] Kosek E Ekholm J Nordemar R A comparison of pressure pain thresholds in different tissues and body regions. Long-term reliability of pressure algometry in healthy volunteers Scand J Rehabil Med 1993 25 117 124 8248762

[B65] Ginya H Asahina J Yoshida M Segawa O Asano T Ikeda H Hatano YM Shishido M Johansson BM Zhou Q Development of the Handy Bio-Strand and its application to genotyping of OPRM1 (A118G) Anal Biochem 2007 367 79 86 10.1016/j.ab.2007.04.052 17570330

[B66] Glamsta EL Morkrid L Lantz I Nyberg F Concomitant increase in blood plasma levels of immunoreactive hemorphin-7 and beta-endorphin following long distance running Regul Pept 1993 49 9 18 10.1016/0167-0115(93)90378-L 7904083

